# Development of a family-based treatment for co-occurring intimate partner violence and child maltreatment: the MST-IPV model

**DOI:** 10.1192/bjb.2023.103

**Published:** 2025-04

**Authors:** Cynthia Cupit Swenson, Cindy M. Schaeffer

**Affiliations:** 1 Medical University of South Carolina, South Carolina, USA; 2 University of Maryland, Maryland, USA

**Keywords:** Post-traumatic stress disorder, psychosocial interventions, trauma and stressor-related disorders, childhood experience, cognitive–behavioural therapies

## Abstract

Intimate partner violence (IPV) is a significant global problem that affects the health of children, parents/caregivers and extended family. The effects can be lifelong and span generations. Treatments for IPV are focused largely on individual work with men as the primary aggressor. Even when the situation includes child maltreatment, generally all family members are referred to a host of providers for varied treatments. Traditionally, couples and family work does not occur. In this article, we detail the development and practice of a comprehensive treatment model for complex cases of co-occurring IPV and child maltreatment that is inclusive of the family and couple. Of particular note, the development of this model, Multisystemic Therapy for Intimate Partner Violence (MST-IPV), involved input from the IPV stakeholder community.

Intimate partner violence (IPV) is a serious global problem affecting millions of people annually. Globally, approximately 27% of women and girls over 15 years of age have experienced physical or sexual IPV. Rates are higher in some countries than others.^1^ In the USA, 28% of women experience physical IPV and 26% experience sexual IPV in their lifetime.^2^ In the year ending March 2022, 5.0% of individuals over 16 years of age in England and Wales experienced domestic abuse; 6.9% were female and 3.0% were male.^3^

The impact of experiencing and witnessing IPV is far-reaching across all family members, extended family and the ecology, and can be carried forward for generations.^4^ The ecology includes individuals who play a significant role in the life of the family, such as friends, neighbours, co-workers, classmates or others specific to a given family. Those who directly experience IPV often suffer lasting and harmful effects to their emotional, physical and economic well-being.^5^ IPV recipients experience lower quality of health, and higher rates of depression, anxiety, post-traumatic stress disorder (PTSD), suicidal ideation and attempts, and substance misuse.^6^ They are at risk of experiencing severe health problems such as heart disease, autoimmune disorders and cancer.^6^

For children, witnessing IPV may involve seeing or hearing violence between two caregivers or knowing about it through some other means, such as seeing a caregiver injury or being told that IPV occurred.^7^ Globally, the 275 million children that witness IPV annually^8^ are at risk of lifelong health and mental health difficulties. Experiencing IPV in addition to physical abuse is called the double whammy, and relates to an increased risk of becoming involved in the criminal justice system and showing aggressive, violent behaviour.^9^

Given the high prevalence and the serious, long-term and intergenerational impact of IPV, making comprehensive and effective treatments available for children, adults and families is critical. However, development, research and dissemination of family-targeted prevention and comprehensive treatments have not kept pace with an understanding of prevalence and impact.^10^

## Commissioning a new IPV model

In an effort to address increasing numbers of cases involving IPV and child abuse/neglect, child protection services in Connecticut, USA, which is called the Department of Children and Families (DCF), commissioned the authors to develop a new model to treat the families they serve. At the time of the commissioning, the services available for IPV in Connecticut would require a family to go to multiple providers. IPV services for male primary aggressors were mainly group-based and educational. Shelter services were available for women, and counselling was part of the shelter programme. Individual services were available for children and adults, but for specialised care (e.g. trauma, substance misuse, couples and family conflict) each family member would need to see multiple different providers for different issues. These providers were mainly state mental health, private clinics, private practice, psychiatric or substance misuse hospital programmes or residential care. They rarely collaborated as they were from different programmes and so services were siloed. DCF became involved when there was a report of maltreatment or threat of harm because of IPV. Their role was to investigate, broker services for families and monitor safety and progress. DCF was not a treatment provider *per se*. With regard to child maltreatment more specifically, at the time of the commissioning, a treatment model based on Multisystemic Therapy for Child Abuse and Neglect (MST-CAN)^11^ had been implemented successfully in Connecticut for 14 years in a specialised substance misuse programme called MST-Building Stronger Families.^12^ DCF was favourable to expanding this model for families experiencing IPV, but MST-CAN was not specialised for IPV. Specifically, DCF sought a model that was comprehensive (to serve all family members), could treat their most serious IPV cases and could meet the needs of families in which IPV was occurring but parents wanted to retain their relationship. More specifically, DCF was seeking an approach that would end IPV violence, prevent child out-of-home placement, improve mental health of family members, and keep couples and families together if safe to do so and if the couple desired to stay together. In addition, a particularly critical identified gap was for services that could help reduce the risk of post-separation violence for those partners who chose to end their relationship, given that violence often continues during and after relationship termination.^13^ Moreover, a need was identified for couples sharing children to co-parent and navigate custody and visitation arrangements post-separation.

The purpose of this article is to describe the development of a new treatment model that would be family-based and address the co-occurring concerns of IPV and child physical abuse and/or neglect among families involved in the child protection system. MST-CAN was considered a starting point for the maltreatment, but additional interventions would be needed to treat IPV. The development of this model was carried out through a series of steps, including (a) listening exercises with key stakeholders, (b) review of the scientific literature on treatments for IPV and risk factors that indicate potential treatment needs, and (c) combining the information learned from the above two steps to delineate a model that is vastly different from traditional outpatient models for use in clinical practice for families experiencing IPV and maltreatment. It should be noted that the model developers did not approach this work with the expectation that they already understood what the full model would entail.

## Ethics statement

This manuscript consists of review of a DCF report on listening sessions conducted by DCF and the scientific literature. The individuals who participated in the DCF listening sessions and whose responses were included in the report were de-identified. As the information reviewed was from a DCF report and not research, consent was not required and an ethics review was not needed.

## Step 1: stakeholder perspectives, listening exercises with groups and individuals

DCF leadership determined that listening to the community to understand their viewpoints was a first step. To design a model that would fill existing gaps in service, be consistent with the risk factor literature and be locally feasible and acceptable, it was critical to gather the viewpoints of stakeholders in Connecticut who work with or who are affected by IPV. This was conducted through individual and group listening exercises. It was apparent early on that the terms ‘perpetrator’, ‘offender’ or ‘victim’ were problematic for stakeholders. These terms stem from an individualistic and criminal framework, rather than from a more compassionate, contextual, systemic and behavioural health framework. Accordingly, for the remainder of this article and in our own work, we use the term ‘primary aggressor’ to refer to the person who commits IPV, thereby focusing exclusively on the behaviour itself (partner aggression) rather than invoking stigmatising criminal connotations. For cases of mutual/bidirectional partner violence, the term is easily modified to simply ‘aggressors’. Similarly, the person(s) against whom IPV has been enacted is more than a ‘victim’, a term that can feel disempowering and minimise the partner's ability to be part of solutions to family violence. ‘Recipients of aggression’ – the term we will use going forward – makes space for the view that recipients are strong and capable even though they have been harmed by the dangerous and inappropriate acts of violence and psychological aggression conducted toward them. We are indebted to the Connecticut stakeholder community that contributed this alternative language to model development.

### Procedures

Interview questions were finalised by DCF state leadership. The goal was to obtain information about the real-life experiences regarding acts of IPV itself, experience with current interventions available in Connecticut and perceived gaps in services. All participants were invited by the DCF central office or a DCF area (regional) office to attend group or individual listening sessions. Group listening exercises were conducted in two DCF area offices and at the Connecticut Center for Nonviolence in Hartford, during autumn 2015. Individual listening exercises were conducted in three separate DCF offices. After all listening exercises were completed, the comments were reviewed for consistent themes.

### Participants of listening exercises

Community stakeholders (*N* = 47) from various regions in Connecticut participated in five group and individual listening exercises, with oversight by a state-level DCF manager and observation by the authors. Most participants were professionals that dealt with IPV cases (e.g. IPV specialists, programme managers and directors, father advocates, victim advocates, court advocates, representative from the Attorney General's office). A total of 60% were female. Two mothers (recipients of aggression) participated individually and two fathers (primary aggressors) participated in a group listening session. These four had prior open cases with DCF and were invited by the regional office that they had worked with in the past. Neither group nor individual listening sessions included primary aggressors or recipients of aggression with currently open DCF cases.

### Results of listening exercises

Table 1 shows the themes and consensus statements related to those themes. Participants offered their ideas about characteristics and clinical presentation of family members who experience IPV, risk factors, treatment components that would be effective, gaps in current interventions in their area, outcomes that should be considered as success of IPV treatment and advice to therapists working with families experiencing IPV. Collectively, stakeholders’ comments suggested a comprehensive treatment model that would treat all family members.

**Table 1:** Results of the listening exercises with key stakeholders

Theme	Consensus
Characteristics and clinical presentation of family members who experience IPV	Current untreated mental health issues (anxiety, depression, aggression, substance misuse, developmental disabilities) History of trauma (experiencing IPV in childhood or other trauma) Vulnerability among undocumented persons or immigrants (low social support, language barriers, cultural differences, financial challenges)
Stakeholder-reported risk factors for IPV	Substance misuse Mental health difficulties Cognitive limitations History of childhood physical or psychological abuse History of witnessing violence Vulnerability among transgender and same-gender couples
Gaps in current interventions for IPV in Connecticut	Multiple treatment needs requiring multiple providers (at least six) that make attending treatment challenging Lack of transportation to attend treatment No available affordable childcare Few services available and do not meet the family needs (e.g. short-term anger management group for the primary aggressor) Current treatments available do not have an understanding of how to manage IPV Some treatments open to men only Some programmes work with women only because of an ‘old school’ view of IPV Most programmes do not address mutual violence Lack of services for non-English-speaking families Services have strict time limits No intensive in-home treatment available that focuses on the primary IPV recipient, the primary aggressor and children simultaneously A lack of comprehensive services provided by one treatment provider to address all clinical facets of IPV No services that focus on helping the couple stay together if safe and they desire to stay together
Treatment components for IPV needed to promote successful outcomes	Comprehensive assessment to identify the origin of IPV Identification and treatment for substance misuse Treatment for mental health challenges Education on how IPV affects children and the family Case management to help empower independence for the IPV recipient Practical needs, such as housing, employment, budgeting, education, childcare Therapist should increase awareness of different cultures, beliefs and norms of clients, and laws and policies related to IPV
Successful outcomes for families experiencing IPV	Stopping the occurrence of IPV Co-parenting without violence Parental acknowledgement of the impact of IPV on children and partners Children's progress and success in school (e.g. attendance, improved grades, less aggression) Helping children understand that violence is not normal Healing of past traumas Helping the family stay together safely
Advice for therapists working with a family experiencing IPV	Focus on safety and protection of children Keep parents focused on health and well-being of their children Remain open minded, non-judgemental Remember that treatment will need to be individualised Help reduce a family's social isolation Be aware that women who leave their partner generally go back Understand that if the couple breaks up they still must co-parent without violence Include both partners in treatment to help them learn to solve conflicts Do not push couples to break up; they must decide Keep in mind the relationship between the family and child protection is very important; work with them to come in gentler For the sake of collaboration and communication, provide regular progress reports to child protection

IPV, intimate partner violence.

## Step 2: scientific literature on IPV that informed model development

As noted earlier, step 2 involved a review of the scientific literature to understand what would be needed in a treatment model to sufficiently address the family's needs regarding IPV. MST-CAN was a model starting point to address the maltreatment. The literatures reviewed were on existing effective treatments and risk factors for IPV that indicate potential treatment needs.

### Existing and effective treatments for IPV

Existing services for IPV tend to be fragmented and victim-oriented, rather than integrated and family-oriented. IPV is commonly viewed as a problem imposed on a female victim by a male offender. Typically, adults who experience IPV are offered individual mental health treatment and advocacy services oriented toward helping them separate from the IPV aggressor. Child witnesses to IPV may or may not also be referred for individual mental health treatment. If aggressors are referred or mandated to treatment, the treatment is usually in a group format with other IPV aggressors, and its helpfulness to the individual and family is uncertain at best. Although a few specified models for treating primary aggressors now exist, there is little evidence that these models are effective in preventing future incidents of IPV.^14^ With regard to the partner relationship, this individually oriented approach runs counter to the research literature on IPV: (a) a large portion (45–95%) of IPV does not fit a traditional ‘victim–offender’ profile, and instead involves mutual aggression;^15^ and (b) couples experiencing IPV have separation rates comparable to, not higher than, couples in nonviolent relationships.^16^ Couples that stay together need treatment to help them prevent escalation and conflict.

### Effective IPV treatment for partners: domestic violence focused couples therapy

Despite the need for partner intervention, IPV treatment models have not focused on the couple's relationship. One exception is the work of Stith and colleagues^15^ on a model for couple's work that is called Domestic Violence Focused Couples Therapy (DVFCT).

#### Description of DVFCT

The DVFCT model consists of an assessment and two major phases. The assessment phase involves use of formal assessment instruments, individual interviews and a lethality assessment to determine if the couple is a good fit for the treatment and if there is any danger to either partner if they were to speak in treatment about their situation. Couples are excluded from this model if either is fearful of violence from anything that might be said in conjoint therapy, if there is a significant discrepancy between the partners in the IPV specifics that occurred, if either partner has a serious untreated substance misuse problem, if there is an unwillingness to remove weapons from the home and if the partners desire to dissolve their relationship.

Phase 1 of treatment involves five sessions, in which the partners meet with the therapist individually. In the separate sessions they work on telling their story, paying attention to early signs of change, determining what they and others would see if their relationship made a drastic change (the miracle question). They learn what qualities they would look for in a healthy relationship and how those could show up in their relationship. Psychoeducation components include defining types of abuse, learning about the cycle of violence and signs of escalation, anger and how it is expressed, practicing meditation and safety planning. Another important topic is managing escalation. For the recipient of aggression, crisis safety plans are made in case there is a need to leave. Each partner is taught a time-out procedure in which they identify their partner's escalation signs. such as red flag situations, red flag words and phrases, and how to use this information to physically and verbally separate themselves in the event that escalation is starting. Safety check-ins are conducted at each session and, if risk or fear is elevated, safety plans are acted on.

In phase 2, the partners move to conjoint sessions where they learn to put negotiated time-out into practice and how to restart conversations without conflict. A great deal of practice occurs on communication strategies and problem-solving of actual situations in their lives. Clients complete DVFCT with specific skills to prevent future acts of IPV.

#### Research outcomes for DVFCT

National Institute of Mental Health-funded research (in the USA) examining DVFCT has studied the model when implemented in either a single-couple format or in multiple-couple groups, all in out-patient settings. Early work on DVFCT showed that IPV recidivism rates were 25% for multiple-couple groups, 43% for single-couple treatment and 67% for no treatment comparison couples. A more recent study^15^ of 83 couples found that the single-couple and multiple-couple group formats led to significant reductions in physical and psychological abuse, but only the multiple-couple group participants experienced reductions in violence, marital conflict, anger and anxiety. Increases in relationship satisfaction were shown for both treatment formats. Given that DVFCT is a couple's therapy, to date, it has not included adult- or child-focused individual treatments (e.g. for trauma).

### Risk factors for IPV, child physical abuse and neglect, and implications for treatment

To develop a comprehensive family-based treatment model, as indicated by the Connecticut stakeholder community, clinicians must understand more than treatment for problematic interactions in the partner relationship. Interventions will be needed to address child physical abuse and neglect, and individual parent and child mental health. Risk factors that set up the conditions for IPV and maltreatment to occur (i.e. driving factors) should be considered. Understanding risk factors that are present in a given family can guide treatment.

### Risk factors specific to child physical abuse and neglect

Many studies examining child physical abuse and neglect have established an aetiology across multiple systems. These include individual child (e.g. aggressive behaviour) and parent/caregiver (e.g. low knowledge of parenting, mental health difficulties, substance misuse), family (e.g. single parent, partner conflict) and social network (e.g. isolation, low participation in community supports).^11^ Likewise, a recent, very thorough systematic review established risk factors in multiple systems that create a context for the occurrence of IPV.^17^ A summary of risk factors for IPV and physical abuse/neglect is shown in Table 2.

**Table 2:** Factors that increase risk for intimate partner violence plus child physical abuse and neglect

Factors that increase IPV risk	Factors that increase child maltreatment risk
Individual	Individual
Alcohol use (greater risk for women)	Aggressive behaviour (child)
Drug use (greater risk for men)	Low knowledge of parenting (adult)
Depression/suicidality	Mental health difficulties (adult trauma, depression, anxiety)
Borderline personality disorder symptoms	Adult substance misuse
Antisocial traits	
Impulsivity	
Trauma symptoms	
Partner relationship	Partner relationship
Marital dissatisfaction (for women)	Partner conflict
Family	Family
Violence in the family of origin	Single parent
Observing parent-to-parent violence (for men)	Family conflict
Observing IPV plus experiencing maltreatment in childhood	
Observing father-only violence	
Situational and contextual factors	Situational and contextual factors
Social isolation	Limited social networks
Low educational attainment	Isolation
Economic instability	Low participation in community supports
Other life stressors	Economic instability
Cultural norms that condone IPV	

IPV, intimate partner violence.

### IPV risk factors specific to mental health difficulties

Drug and alcohol use are consistent risk factors among adults. Rates range from 40 to 92% across studies.^18^ Interestingly, alcohol use has been found to be a stronger predictor of carrying out IPV for female aggressors and drug misuse is a stronger predictor for men carrying out IPV.^17^ In addition, marital dissatisfaction is a factor in female, but not male offending.^15^

Mental health difficulties, including depression/suicidality, borderline personality disorder symptoms, antisocial traits and impulsivity, all have been found to increase risk of committing IPV.^17^ Trauma symptoms are also a likely risk factor for IPV, given strong associations between experiencing the trauma of family violence and later committing it.^19^ It should be noted that although the majority of individuals exposed to IPV in childhood do not become violent, violence in the family of origin increases the risk of committing violence.^20^ That risk increases further when the children also experience other maltreatment. In a recent study of men, experiencing child maltreatment was not linked to later IPV, but observing parent-to-parent violence was associated with an almost threefold increase in the odds of carrying out IPV.^19^ Individuals who experienced a combination of observing IPV and experiencing maltreatment were more than four times more likely to commit IPV in adulthood, compared with individuals who had no exposure to violence in childhood. In addition, type of IPV witnessed was also predictive. The risk of committing IPV in adulthood increased fivefold when the IPV witnessed was bidirectional, whereas observing father-only violence was associated with nearly a threefold increase, and observing mother-only IPV did not predict committing IPV in adulthood.^20^ Situational and contextual factors also contribute to committing IPV. Social isolation, low educational attainment, economic instability, other life stressors and cultural norms all increase risk.^17^

## Treatment implications of a multidetermined aetiology

As indicated by the literature on aetiology, the risk of child maltreatment plus IPV is related to multiple factors across multiple systems. When IPV and child maltreatment co-occur, a family-oriented, comprehensive and integrated treatment model is needed to address all systems in which risk occurs. At this time, there is no known research-supported model that fits this comprehensive need.^10^ To fill this gap and meet the requirements of the DCF request to develop a new model, the authors combined knowledge learned from the listening sessions and the research literature with knowledge from 20 years of rigorous research and dissemination of MST-CAN. This particular complex treatment model (MST-CAN) is being implemented with success in the USA, Australia, Norway, The Netherlands, Switzerland and England. It has been successfully implemented in England for 10 years. The authors determined that the knowledge attained from the present project supported the use of MST-CAN for maltreatment, but the model does not address IPV. The literature indicated DVFCT is an evidence-based treatment for couples work. The authors met with Dr Stith and attained her permission to use DVFCT with MST-CAN. The blended model was named Multisystemic Therapy for Intimate Partner Violence (MST-IPV). Stith and colleagues^15^ were a resource used in conjunction with the MST-CAN materials to develop training resources for the team. DCF agreed to pay for the implementation of the clinical team and a small, quasi-experimental pilot study that is currently underway. The 1-year model development work was funded by the Annie E. Casey Foundation. This model is the first implementation of DVFCT using a home-based delivery model and including broader individual and family intervention components. It is also the first implementation of an MST-CAN-based model for families experiencing severe IPV. Next, we describe the MST-IPV model.

## Description of MST-IPV

**Fig. 1:**
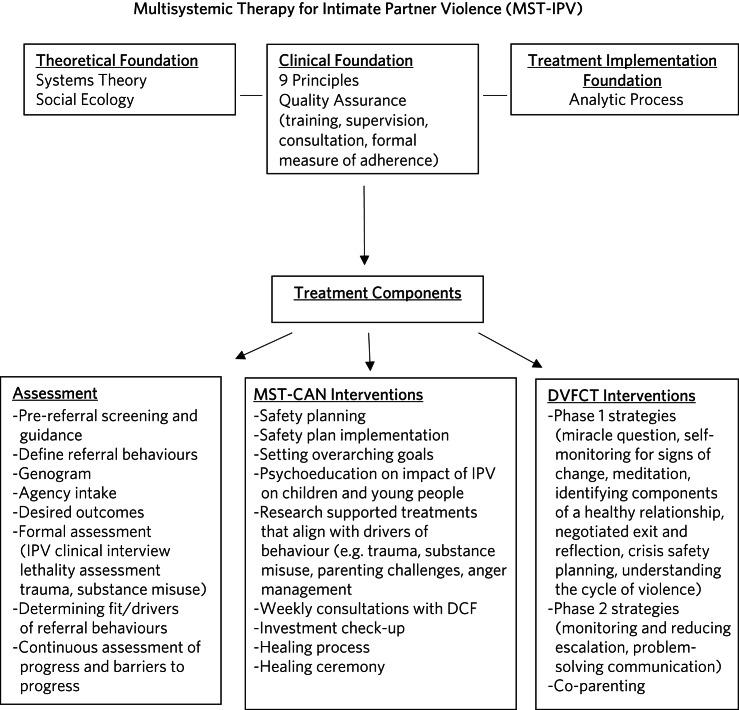
Summary of the Multisystemic Therapy for Intimate Partner Violence model. DCF, Department of Children and Families; DVFCT, Domestic Violence Focused Couples Therapy; IPV, intimate partner violence, MST-CAN, Multisystemic Therapy for Child Abuse and Neglect.

In this section, we describe the blending of the two treatments (MST-CAN and DVFCT) and note any additions or changes to each model to meet the needs of families experiencing maltreatment and IPV. See Fig. 1 for a summary of the model. The MST-CAN model^11^ is based on standard multisystemic therapy (MST), developed by Henggeler and colleagues for delinquent youth.^21^ The core of MST used in all adaptations with populations other than delinquent youth, includes an ecological theoretical foundation, a clinical foundation that is represented by nine principles and a treatment implementation foundation including an analytic process that structures clinical practice.

## MST theoretical foundation

MST is rooted in systems theory^22^ and social ecological models of behaviour.^23^ As such, the child is viewed as embedded in multiple systems (parent, family, extended family, school, peers, community) that have direct and indirect influences on their behaviour. The influences from all systems are considered to be reciprocal and bidirectional. That is, children have some influence on each system and are influenced by each system. Systems that are closer to the child and with which they have more contact (i.e. parents, siblings) have stronger influence. The implications of multiple systems influencing the child and family is that treatment for one system (e.g. parenting) is inadequate for effecting change. Risk factors in all systems surrounding the child must be addressed.

## MST clinical foundation

### Nine principles

The clinical foundation of MST follows nine principles that form a common thread throughout treatment. These principles encompass core characteristics of treatment,^24^ and adhering to them is essential to treatment fidelity that has been associated with clinical outcomes. The principles are shown in Table 3.^24^

**Table 3:** The nine principles of multisystemic therapy

The primary purpose of assessment is to understand the fit between the identified problems and their broader systemic context Therapeutic contacts emphasise the positive and use systemic strengths as levers for change Interventions are designed to promote responsible behaviour and decrease irresponsible behaviour among family members Interventions are present-focused and action-oriented, targeting specific and well-defined problems Interventions target sequences of behaviour within and between multiple systems that maintain the identified problems Interventions are developmentally appropriate and fit the developmental needs of the youth Interventions are designed to require daily or weekly effort by family members Intervention effectiveness is evaluated continuously from multiple perspectives, with providers assuming accountability for overcoming barriers to successful outcomes Interventions are designed to promote treatment generalisation and long-term maintenance of therapeutic change by empowering caregivers to address family members’ needs across multiple systemic contexts

The nine principles are the basis of questions that are part of a monthly phone interview with a parent to assess ongoing fidelity to the model. It should be noted that for the implementation of the MST-IPV model, the basic MST model adherence questions have been supplemented with questions pertaining to implementation of IPV procedures.

## Treatment implementation foundation

### The analytic process

The MST analytic process serves as a roadmap for determining the focus and sequencing of interventions. Underlying the process is ongoing efforts by the clinical team to engage families and recognise their culture and strengths. The process begins with attaining well-defined and measurable referral behaviours (e.g. parent hitting their partner and leaving bruises). Second, all members of the family and pertinent ecology members are interviewed to gather their desired outcomes (i.e. goals for treatment). The desired outcomes become the family's overarching goals that, when met, represent the completion of MST-IPV treatment. One of the most critical steps is to determine the ‘fit’ or driving factors for the referral behaviours. Given that MST is an ecological model, driving factors from multiple systems (e.g. parent, family, school, peers) are assessed and the primary drivers are targeted for intervention. For example, a referral behaviour of leaving children home alone and partner violence may have substance misuse as a primary driver. In this case, treatment for substance misuse is prioritised, but the team also assesses the drivers of substance misuse. They may find that substance misuse is an attempt to cope with trauma symptoms. In such case, treatment for PTSD symptoms will also need to be provided by the team. Goals and interventions for the primary drivers are developed and carried out weekly. An ongoing assessment is conducted to understand if the interventions are effective and, if not, then to understand why not and revise. The analytic process is followed throughout treatment.

## Blended treatment components

### MST-CAN

MST-CAN, an intensive family-based approach that has proven effective for families involved with Child Protective Services who are experiencing physical abuse and/or neglect.^11^ Families referred to MST-CAN are those with serious, complex situations. Importantly, the model seeks to capitalise on strengths of all family members, and follows the principle that although the family is having severe problems and facing severe safety risks, the partners, as well as the family, as a whole have the capacity to change. The model has been rated as research-supported by the California Evidence-Based Clearinghouse for Child Welfare,^25^ and is also recommended in the National Institute for Health and Care Excellence guidance for therapeutic interventions after abuse and neglect and the Early Intervention Foundation's Guidebook in the UK.^26^ MST-CAN is currently disseminated across six countries, including England. The primary research was conducted in the USA. In a National Institute for Mental Health-funded, randomised controlled trial (RCT) comparing MST-CAN to enhanced out-patient treatment, evidence of effectiveness across 16 months was shown in four areas. These outcomes include (a) out-of-home placement (significantly fewer children placed and of those placed significantly fewer placement changes), (b) prevention of abuse and neglect (greater reductions in minor assault, severe assault, psychological aggression and neglectful parenting, more likely to use nonviolent discipline), (c) mental health difficulties (greater reductions in adult global psychiatric distress, greater reductions in parent-reported children's internalising symptoms, total behaviour problems, PTSD symptoms, greater reductions in youth self-reported PTSD and dissociative symptoms) and (d) social support (greater increases in total, appraisal and belonging social support). A Swiss study of families who received MST-CAN also found a significant decrease in adult psychiatric distress at 6 months post-treatment.^27^ In addition to clinical outcomes, MST-CAN has proven to be cost-effective. In the USA, $3.31 was saved for each dollar spent.^28^ In England, a study showed £1.59 saved for every £1 spent.^29^

### MST-CAN within the MST-IPV model

This blended model is the first implementation of an MST-CAN-based model for families experiencing moderate-to-severe IPV. It is also the first implementation of DVFCT using a home-based delivery model and including broader individual and family intervention components. First, we will describe more fully the population to be served by MST-IPV. Next, we will detail what has been added or changed to each of the treatments to create the blended model. Also, we will indicate additional interventions that are based on the community listening session feedback.

#### The population served by MST-IPV

The population served by MST-IPV comprises families who have come under the guidance of DCF because of physical abuse and/or neglect of a child plus IPV. The families are otherwise heterogeneous. IPV may or may not have led to an arrest of one or both partners, and protective or no contact orders between the partners and/or toward the children may or may not be in place. The couple may have an intention to remain together or to separate; regardless, in most cases, the couple will both retain custody of the children and will co-parent. Children are in the age range of 0–17 years and many families have multiple children. The primary aggressor and recipient of aggression may be male or female. To serve families who have had recent (rather than only historical) IPV, the report to DCF must have occurred within the past 180 days; multiple prior reports are acceptable. Children may be in placement at the time of referral as long as there is an expectation by DCF that they can be returned to the family once safe to do so (i.e. termination of parental rights is not in process).

Families who are not appropriate for MST-IPV are those with children in out-of-home care with no plan to reunite the family. Families with unsubstantiated maltreatment or IPV reports are not included, to ensure that the model serves the most serious and complex cases. Although families may have experienced historical sexual abuse and need current treatment for PTSD, MST-IPV does not accept cases with current active sexual abuse (i.e. it is not a sex offender treatment).

#### Clinical team

Consistent with MST-CAN, the MST-IPV programme is staffed by a clinical team consisting of a full-time supervisor who carries no case-load, three full-time therapists, and a full-time family resource specialist. To achieve a high level of clinical intensity and to address all identified drivers of IPV and sequelae, each therapist's case-load is limited to a maximum of four families. The family resource specialist assists all families with case management needs, such as budgeting, housing, jobs, drug-free recreation and school needs. In addition, the team is staffed by a psychiatrist or advanced practice registered nurse with prescription privileges to assist with family members who are taking medication for emotional or behavioural difficulties or who have psychiatric crises. The psychiatrist or nurse is available to the team for a 10 to 20% time allocation.

#### Close working relationship with DCF

An important characteristic of the model is the close working relationship with DCF to help mitigate risk of IPV recurrence. The working relationship involves close contact; a role for a DCF employee to gatekeep; a weekly joint consultation; and formal meetings with DCF, the family and the clinical team. The IPV specialist who works for DCF serves as a gatekeeper to refer families and to make sure the service is at full utilisation. This individual also works closely with the MST-IPV supervisor to review cases to determine what kind of family situation is safe enough for home-based services. In Connecticut, the DCF caseworkers are part of a unit that manages only IPV cases. The caseworkers, supervisor and IPV specialist from DCF meet weekly with the team to review the needs and progress of the families, to work together to solve problems and celebrate family successes. This joint consultation is specific to the MST-IPV programme.

In the first month of treatment and every 3 months thereafter, DCF, the MST-IPV clinical team and the family meet together to review progress toward their goals and determine if any shifts need to be made in goals or treatment. This meeting is referred to as the investment check-up. The rationale is that DCF believes in the family and is making a significant investment in them by referring them to MST-IPV, and they are meeting to check up on the investment and how it is going. On a day-to-day basis, the working relationship with the team and DCF cannot be highlighted strongly enough. The idea is for the MST-IPV team, DCF and the family to collaborate on change.

#### Service characteristics

A small case-load of four families for each therapist allows them to provide services to all family members, with the average being five persons per family. Given that families are referred to MST-IPV because of adult behaviour, a special emphasis to reduce risk is on changing adult behaviour. Treatment is delivered at least three times per week in the home, community and places that are safe and convenient for family members, and at times that are convenient for families. In addition, the team operates a 24 h per day, 7 days per week, on-call therapist rotation to help manage crises or safety risks. If, because of safety issues, a child is placed out of the home, services are also provided with the kinship care family or foster family to facilitate rapid return. Many families have court processes that they will be involved in, for which the team provides support.

#### Quality assurance

The purpose of the quality assurance components is to support the clinical team in adhering to the core MST principles and MST-IPV procedures. Adherence to MST principles has proven to be related to positive clinical outcomes.^30^ Quality assurance activities start with trainings in MST, MST-CAN, adult substance misuse treatment, early childhood (age 0–5 years) interventions, DVFCT, and child and adult trauma treatment. In addition, quarterly boosters are held with the team to address areas of need identified by the MST-IPV team. Group supervision with the MST-IPV supervisor and team consultation with an MST-IPV expert are conducted weekly. Importantly, and as noted earlier, the DCF IPV specialist, DCF workers and DCF supervisor attend 45 min of the team consultation weekly, to help with rapid and efficient problem-solving to improve clinical outcomes for families. Finally, as noted earlier, parents or caregivers are interviewed monthly by an independent interviewer, to formally assess model adherence.

#### Pre-referral guidance

As the family's referral is being considered, DCF and the MST-IPV team supervisor complete a screening of the case, and follow a guidance to determine if the information known about the family indicates that the situation is safe enough for treatment to proceed and for the therapists to meet with the couple in the home. If the guidance indicates that the referral should proceed, the MST-IPV supervisor and DCF worker visit the family to explain the treatment and engage them in agreeing to join. This meeting may be with partners separately or together, depending on the situation and any protective orders.

#### Safety focus

Upon the family agreeing to join, a number of safety and assessment strategies assist the team with an understanding of areas of focus for treatment. Before contact with the family, the team works to understand what is known about the event that led to a DCF report and referral behaviours that represent DCF concerns. The team must understand whether there are no contact or protective orders and who those are between and any specific risks to family members, to plan the structure of services.

MST-IPV has an intensive focus on family and child safety for all participants. In the initial session, a family safety plan (from MST-CAN) is completed with the family and DCF caseworker. For the first month, a family safety checklist is completed weekly by a walkthrough of the home with the parent. Any safety issues noted are addressed immediately (e.g. prescription medications left out on a table). Safety plans are changed as new information is attained.

#### Clinical assessment

Once the family agrees to the programme, each member of the ecology (e.g. parent/caregiver, children, grandparents, DCF) is interviewed to understand their views of strengths and needs of the family and their desired outcomes. The desired outcomes identified by the full ecology become the overarching goals to guide the course of treatment (e.g. no new reports of child maltreatment). Next, a more fine-grained assessment is conducted, including (a) a genogram, (b) formal assessment of substance misuse and the occurrence of trauma and trauma symptoms if indicated and (c) assessment of ‘fit’. The fit assessment examines factors that contribute to each of the referral behaviours, with the strongest predictors being prioritised. For example, substance misuse may be a primary driver of the IPV and is targeted for immediate intervention. In addition to these assessments, the team considers whether the family has social supports, and regular assessments of safety are conducted. Importantly, from DVFCT, every partner individually completes an IPV clinical interview that includes a lethality assessment to aid in family safety. Assessment is an ongoing strategy to understand, on a regular basis, interventions that are working or when the clinical work needs to shift to a different driver of the target behaviour. Assessment results are incorporated into the IPV safety plan, which is revised and refined repeatedly throughout treatment.

#### Clinical treatment

Treatments used with families are those that address their risk or driving factors, meet their needs and have research support. Interventions are tailored to the family needs such that not every family receives the same interventions. One exception is that all families complete DVFCT and a healing process and ceremony.

Within MST-IPV, DVFCT can be implemented whether or not the couple is still residing in the same household or plans to remain together, given that co-parenting and child visitation will require use of strategies to prevent violence escalation, regardless of where each person lives. In addition, DVFCT and MST-CAN are not restricted to physical violence. The model also addresses verbal and psychological aggression such as coercion, name calling, put-downs and negative social media posts. Violence and aggression are viewed as a choice of behaviours that can be changed. To effect change, the client and therapist must have an understanding of the ‘fit’ of the behaviour (i.e. what is driving it), the thoughts that start the sequence of abusive behaviours, feelings that move the behaviours forward and where in the cycle they can prevent escalation. Primary aggressors may not recognise coercive behaviours, and will need psychoeducation to recognise and monitor their behaviour.

### Changes in the individual models to facilitate family work

The blended model follows DVFCT's two phase approach, with a few changes. First, the name of the strategy, negotiated time-out, has been changed to negotiated exit and reflect. All clients have families and time-out is a term they are accustomed to hearing regarding parenting of young children. The new name is more reflective of adult (caregiver) behaviour and the expectation that they will negotiate parting from the conflict, reflect on and practice de-escalation, and come back together peacefully. In addition, the negotiated exit and reflect is implemented earlier in treatment than in DVFCT, to help mitigate risk in the home context. A second change is that the IPV crisis plan that details what the recipient of aggression can do to leave if escalation is occurring and they feel afraid (e.g. who to call to pick them up, where to leave keys and papers that might be needed, how to get the children out of the house) is developed very early in treatment. A third change is in psychoeducation. The community listening sessions indicated that it would be important for the parents, and especially the primary aggressor, to understand how IPV affects the children. In response to the community feedback, the authors joined with two artists to develop two booklets on the impact of IPV on children and youth. One is for adults and one is for children/young people. The booklets combine information from the research on the impact of IPV with positive activities that can be carried out in session, such as yoga poses, positive words, West African symbols with various meanings and musical instruments from around the world.^31,32^

As in MST-CAN practice, each family completes the healing process and ceremony. The purpose is for the primary aggressor to take responsibility for their behaviour and apologise to the children and partner, and is based on the abuse clarification literature.^11^ In MST-IPV, one healing letter is written to the recipient of aggression and a second is written to the children. In addition to taking responsibility and fully acknowledging IPV behaviours, the aggressor(s) portion of the healing process (which comes after other treatment goals, such as the elimination of use of violence, have been met) involves changing any remaining unhelpful cognitions related to their aggression through writing a letter. The primary aggressor is given an outline as to what should be addressed in the letter. After it is written, there may be several drafts to exclude language that may lead family members to feel blamed or at fault for the IPV or any other behaviour that is included in the apology. Individuals that have challenges with writing may speak their letter orally and have it typed on laptop by the therapist, and it is then edited from the hard copy. In the healing ceremony, the primary aggressor reads the letter to the family and gives the apology. Family members comment or ask questions. It has been the authors’ experience that the healing ceremony is highly valued by the family and sets the stage for more positive interactions.

Aside from safety strategies, DVFCT and the healing process/ceremony, other treatments depend on the drivers of the maltreatment or IPV and behaviours for which interventions are needed. Common research-supported treatments provided, depending on family need, include cognitive–behavioural therapy for anger management,^33^ adult^34^ and child^35^ trauma treatment, reinforcement-based treatment for adult substance misuse,^36^ behavioural family therapy focused on problem-solving and communication,^37^ behavioural parent training interventions and school-based interventions (e.g. for school refusal or behaviour management).^21^ Through the family resource specialist, case management is provided to families on budgeting, health needs, housing, finding employment, drug-free recreation and dealing with legal system issues. Underlying all treatment is respect for the family's culture, work to keep families engaged, great valuing of the relationship with DCF and high engagement of ecology members (e.g. extended family).

### Replicability

The MST-IPV model is quite complex and includes multiple treatments for multiple people with serious needs and strong monitoring of safety. The question may arise as to whether this model will be replicable in other sites. Given the work that has taken place on MST for over 30 years and MST-CAN for 20 years with complex situations and clients with serious clinical and safety needs, the likelihood of replicability with fidelity is very high. Major strengths of the pilot and the MST-IPV model that will support replicability are lessons learned from 5 years of successful implementation of MST-IPV that features a 100% treatment completion rate. In addition, MST implementation characteristics that foster successful outcomes used in MST-IPV are the analytic process, nine principles, the quality assurance process and use of evidence-based treatments. MST-CAN has been successfully implemented in six countries with many different cultures, including families on refugee status from Middle Eastern and African countries. Successful implementation has taken place in England for 10 years. The main factor that will need consideration is the relationship with DCF and determining how systems can be structured to support the needed level of interaction and collaboration.

In conclusion, MST-IPV was developed at the behest of DCF in Connecticut in the USA. DCF provided oversight of group and individual listening exercises that represented the views of what is needed in treatment from community stakeholders interacting with IPV. The authors took that information and considered it in conjunction with a review of the scientific literature that indicated the effectiveness of DVFCT and their own work using MST-CAN, a family-based treatment for physical abuse and neglect. The result is a family and ecological model based on blending MST-CAN and DVFCT. This model serves the partners, children, family and ecology. The overarching goal is to eliminate IPV while keeping families together if it is safe to do so and if partners want to be together. The treatment focuses heavily on safety and clinical interventions to eliminate IPV and child maltreatment, improve adult and child mental health, reduce substance misuse if it is occurring and solidify natural social supports. This model is the first to provide DVFCT in the home, and is the only known home-based approach to address IPV. The MST-IPV model has been successfully implemented in Connecticut for 5 years, and is currently being evaluated in a quasi-experimental research trial and a qualitative study there. Although the implementation has been in the USA, given that MST-CAN (a large part of the model) has been successfully implemented in multiple countries with multiple cultures for the past 20 years, the model should be adaptable to other countries. In addition, the authors’ hope is that this article will inform clinical practice for anyone who is treating families where IPV has occurred.

## Data Availability

Data availability is not applicable to this article as no new data were created or analysed in this study.

## About the authors

**Cynthia Cupit Swenson,** PhD, is Professor of Psychiatry and Behavioral Sciences at the Division of Global and Community Health, Medical University of South Carolina, South Carolina, USA. **Cindy M. Schaeffer,** PhD, is Associate Professor of Psychiatry at the Division of Child and Adolescent Psychiatry, University of Maryland, Maryland, USA.
